# CGO and SNS Optimization Algorithm for the Structures with Discontinuous and Continuous Variables

**DOI:** 10.1155/2022/4211707

**Published:** 2022-12-06

**Authors:** Amin Ghannadiasl, Milad Zarbilinezhad

**Affiliations:** Department of Civil Engineering, University of Mohaghegh Ardabili, Ardabil, Iran

## Abstract

This study aims to find discontinuous and continuous approaches to reducing the size of planar truss structures with a specified shape and topology. The member's section area has assumed to be a decision variable, and the objective function is to minimize their weight. The member stresses and node displacements are the constraints that must maintain within the allowed limits for each condition. Chaos game optimization (CGO) and social network search (SNS) algorithms were used to optimize four well-known planar truss structures. In discontinuous-size cases, the results of the social network search (SNS) algorithm are the most cost-effective. However, the results of the chaos game optimization (CGO) algorithm are the most cost-effective in continuous-size cases.

## 1. Introduction

Developing a material structure that bears loads optimally is defined as structural optimization [1]. In engineering design, for example, the primary goal is to comply with basic standards while simultaneously achieving the best economic outcomes, i.e., selecting design parameters that meet all design criteria at the lowest possible cost. Optimization is a method for resolving problems like these. Commonly, the goal of structural optimization issues is to minimize objective function (typically the structural weight). This minimal design's value is limited to various problems based on factors such as displacements and stresses, as well as the structural member's minimum dimensions or cross-sectional areas. The optimization problem may be classified into two types according to whether the variables are continuous or discontinuous [2, 3]:

- Discontinuous optimization is an optimization problem considering discontinuous variables in which an object, such as an integer, must be found from a countable set.
- A continuous optimization problem is one in which an optimal value must obtain from a continuous function. It might be limited or multimodal.

In recent decades, the ubiquity of trusses as structural systems has made their optimization an important engineering endeavor. Design ideas due sustainable, reliable, and cost-effective for the structure have been the impetus for the many developments of optimization procedures. Therefore, a trustworthy optimization strategy is always needed to tackle a structural optimization issue. Metaheuristic approaches are generic optimization tools that do not need a continuous objective function(s) or gradient information to direct the search process in this respect. Because of this positive quality, researchers in several sectors of science and engineering employ these approaches extensively [4–9]. Metaheuristics are also often applied to handle structural optimization problems, and studies have shown that these algorithms are effective in these situations [10–15]. In discontinuous design variables, most known optimization approaches are ineffective because they interpret design variables as continuous. To deal with the discontinuous character of design variables, a few mathematical programming-based methodologies have been developed [2, 3]. On the other hand, in real-world structures, the number of members, the degrees of freedom for every node, and the stiffness matrix can compound the problem optimization. Therefore, in truss optimization, meta-heuristic algorithms are more desirable because they can solve problems in fewer structural analyses. The genetic algorithm (GA) and other novel heuristic algorithm-based optimization methodologies were proposed to achieve optimized designs for discontinuous structural systems because few mathematical programming technique-based approaches considered design variables as discontinuous. Many researchers have looked at GA-based optimization approaches, including Hajela and Lee [16], Rajeev and Krishnamoorthy [17], Camp et al. [18], Wu and Chow [19], Erbatur et al. [20], Pezeshk et al. [21]. In addition, various other studies are conducted in the field of structure size optimization. Graph-based parameterization concept was employed by Giger and Ermanni [22]. Democratic PSO (DPSO) was used by Kaveh and Zolghadr [23]. A harmony search (HS) and a firefly algorithm (FA) were put to the test by Miguel and Fadel Miguel [24]. Also, Tejani et al. assessed the symbiotic organisms search (SOS) algorithm for structural optimization [25–29]. The results show that the proposed adaptive SOS algorithm [25] is more efficient and reliable for the optimization of the structures under dynamic excitation. In the continuation of their research, a multiobjective adaptive symbiotic organisms search (MOASOS) is applied to truss optimization [27]. In this study, the weight of the truss and nodal displacement are objective functions. The results of the MOASOS algorithm demonstrated that this algorithm can provide a competitive and better result than the previous studies. Also, structural optimization using the multiobjective plasma generation optimization (MOPGO) algorithm is studied by Kumar et al. [30].

A hybridized CSS and a sizable bang-huge crunch (CSS-BBBC) were introduced by Kaveh and Zolghadr [31]. The adaptive ground finite elements technique was introduced by Noilublao and Bureerat [32]. Truss constructions with discrete variables were optimized using the mine blast algorithm (MBA) by Sadollah et al. [33]. The particle swarm optimizer (PSO), the heuristic particle swarm optimizer (HPSO), and the particle swarm optimizer with a passive congregation (PSOPC) were used by Li et al. [34, 35] for the optimum design of pin-connected structures. Ho-Huu et al. [36] developed an adaptive elitist differential evolution (AEDE) to optimize a truss with discrete design variables. The centers and force formulation (CAFF) was used by Farshi and Alinia-Ziazi [37] for the sizing optimization of a truss. An artificial bee colony (ABC) method was utilized by Hadidi et al. [38] to optimize the planar and space trusses. Eskandar et al. [39] used the water cycle algorithm (WCA) for the structural optimization of planar trusses. For size optimization of truss structures, Sangtarash et al. [40] presented a method known as the hybrid artificial physics optimization and big bang-big crunch algorithm (HPBA). Kooshkbaghi and Kaveh [41] proposed the artificial coronary circulation system algorithm (ACCSA) for sizing optimization of truss structures with continuous variables. An evolutionary algorithm based on the hybrid GA and PSO (HGAPSO) was developed by Kaveh and Malakoutirad [42] to solve force method-based simultaneous analysis and design problems for frame structures. An optimality criterion method (OCM) was used by Khan et al. [43] for large-scale structures. Yang et al. [44] proposed the computational efficiency of accelerated particle swarm optimization combined with different chaotic maps for global optimization (CPSO3) with continuous and discrete variables. Azad and Hasancebi [45] presented an elitist self-adaptive step-size search (ESASS) for optimal sizing of truss structures based. The enhanced colliding bodies optimization (ECBO) was introduced by Kaveh and Ilchi Ghazaan [46] for sizing optimization of truss structures with continuous and discrete variables. Talatahari and Azizi [47] have introduced a novel meta-heuristic approach termed the Chaos Game Optimization (CGO) Algorithm. In addition, Bayzidi et al. [48] recently introduced a novel meta-heuristic technique called Social Network Search (SNS). The CGO algorithm's fundamental premise is based on certain chaos theory concepts. The chaos game concept and fractal self-similarity difficulties are considered. The SNS algorithm replicates social network users' efforts to achieve more significant popularity by modeling choice emotions in expressing their thoughts. The mixed continuous/discrete engineering optimization problems have been effectively solved using CGO and SNS, although they are fundamental methods. On the other hand, Singh et al. [49] present an improved version of the follow-the-leader (iFTL) algorithm for the optimization of the truss problems.

This paper aims to determine the optimal discontinuous and continuous sizes for planar trusses of a certain design and topology so they may be constructed. It is necessary to conduct some case studies, both discontinuous and continuous in truss size, to assess the efficacy of each technique. For this aim, the very recent CGO and SNS algorithms are used in this paper. The CGO and SNS algorithms will each be broken down and examined in further detail in the following sections. Finally, following the introduction of the trusses and the creation of the truss design optimization model, the outcomes of the two approaches are exhibited and compared with the previous study's conclusions. The following is how the remainder of the paper is organized: in Section 2, the optimization problem is defined. In Section 3, the concepts of CGO and SNS are briefly described. The continuous and discontinuous design variables used in CGO and SNS to optimize truss structure size are discussed in Section 4. The CGO and SNS algorithms are used to optimize four well-known truss designs in this section, with the results contrasted in discontinuous and continuous design variables. Finally, Section 5 provides a brief conclusion of the present study.

## 2. Description of the Truss Optimization Problems

The objective of truss size optimization is to lower the structure's weight while adhering to stress and deflection restrictions. As design variables, cross-sectional areas are chosen from a list of the allowable sections. The problem can be formulated as follows:

(1) $$\begin{array}{c} Minimize:W\left(\left\{X\right\}\right)=\overset{z}{\underset{i=1}{\sum }}\rho_{i}\cdot X_{i}.L_{i}. \end{array}$$

Subjected to:

(2) $$\begin{array}{c} \sigma_{\min}\leq \sigma_{p}\leq \sigma_{\max},p=1,2,\ldots ,z\text{,}\\ \delta_{\min}\leq \delta_{j}\leq \delta_{\max},j=1,2,\ldots ,n\text{,}\\ X_{p}\in allowable\;section\text{,} \end{array}$$

where the members of cross-sectional areas, denoted by the vector *X*, are taken into account (design variables), *ρ*_*i*_ is the material density, *W*({*X*}), and L_*i*_ and X_*i*_ are the weight of the truss, the length of members, and the cross-sectional area, respectively. z is the number of members, and n is the number of nodes. The bottom displacement limit is denoted by *δ*_min_, the upper displacement limit is represented by *δ*_max_, and the displacement of node *j* is represented by *δ*_*j*_. Furthermore, the bottom limit of stress is represented by *σ*_min_, the upper limit stress is represented by *σ*_max_, and the stress of member *p* is represented by *σ*_*p*_. *X*_*p*_ is the cross-sectional area of the member p that is considered from the allowable section. The components in the allowable section list are ordered in ascending order.

For unconstrained optimization problems, CGO and SNS are presented. A penalty function method is used to deal with restrictions. In this method, the value of the objective function is determined by the sum of the constraint violation in this technique, and the constrained optimization problem is transformed into an unconstrained one using the following formula:

(3) $$\begin{array}{c} P={\left(1+\xi_{1}\cdot \left(\overset{K}{\underset{j=1}{\sum }}\;\Phi^{j}\right)\right)}^{\xi_{2}}\times W\left(\left\{X\right\}\right)\text{,} \end{array}$$

where *P* is the unconstrained objective function, and *ξ*_1_ and *ξ*_2_ are the penalty function coefficients. In this study, *ξ*_1_ and *ξ*_2_ are both set to 1. *K* is the number of constraints, and Φ^j^ is the number of constraints connected to *j*^th^ constraint:

(4) $$\begin{array}{c} \Phi^{j}=\left\{\begin{array}{cc} \left|\frac{c_{\max}^{j}-c^{j}}{c_{\max}^{j}}\right|\text{,} & ifc^{j}>c_{\max}^{j}\text{,}\\ \left|\frac{c_{\min}^{j}-c^{j}}{c_{\min}^{j}}\right|\text{,} & ifc^{j}<c_{\min}^{j}\text{,}\\ 0\text{,} & otherwise\text{.} \end{array}\right. \end{array}$$

The lower and upper boundaries of the *j*^th^ constraint are *c*_min_^*j*^ and *c*_max_^*j*^, respectively, and the value of the *j*^th^ constraint is *c*^*j*^.

## 3. Concepts of Chaos Game Optimization (CGO) and Social Network Search Algorithm (SNS)

### 3.1. CGO

Talatahari and Azizi [47] proposed the CGO algorithm. The configuration of fractals utilizing chaos game approaches, as well as fractal self-similarity challenges, is examined in this program, which is based on chaos theory notions. The CGO method is mathematically modeled using the fundamental concepts of fractals and chaos games. This mathematical model is predicated on the assumption that to complete the overall form of one, it is necessary to build several Sierpinski triangles by making use of seeds that are allowed to exist inside the search space. In this particular scenario, the production of fresh seeds inside a Sierpinski triangle is also used. Three seeds are used to create a temporary triangle for each qualifying seed in the search area (*X*_*i*_) [47]:

- The current position of the global best (GB)
- The mean groups (*MG*_*i*_), current position
- The itch solution candidate's (*X*_*i*_) position as the chosen seed

The following is the procedure for the first seed:

(5) $$\begin{array}{c} {Seed}_{i}^{1}=X_{i}+\alpha_{i}\times \left(\beta_{i}\times GB-\gamma_{i}\times MG_{i}\right),i=1,2,\ldots ,n\text{,} \end{array}$$

where *X*_i_ is the *i*^th^ solution candidate, GB is the global best so far discovered, and *MG*_*i*_ is the average of some picked eligible seeds. Each of the *β*_*i*_ and *γ*_*i*_ represents a random number of 0 or 1 for modeling the option of rolling a dice. At the same time, *α*_*i*_ is the randomly generated factorial for modeling the mobility constraints of the seeds.

The second seed can move in a manner that is analogous to the movement of the first seed in the direction of a point that is located on the connecting lines between the *X*_*i*_ and the *MG*_*i*_. Despite this, the movement of the second seed is restricted by some factorials that were generated at random. The following is the presentation of the second seed:

(6) $$\begin{array}{c} {Seed}_{i}^{2}=GB+\alpha_{i}\times \left(\beta_{i}\times X_{i}-\gamma_{i}\times MG_{i}\right),i=1,2,\ldots ,n\text{,} \end{array}$$

where *α*_*i*_ represents a random number of 0 or 1 for modeling the option of rolling a dice, and each of the *β*_*i*_ and *γ*_*i*_ represents a random integer of 0 or 1 for modeling the mobility constraints of the seeds. The remaining criteria are the same as for the first seed.

*MG*_*i*_ came in third place. A random number-generating function that only produces two integers, 0 and 1, is used to demonstrate this. It is worth mentioning that the seed may migrate toward the connecting lines, *X*_*i*_ and *GB*. Several random factorials, such as the following, may alternatively be used to accomplish this goal:

(7) $$\begin{array}{c} {Seed}_{i}^{3}=MG_{i}+\alpha_{i}\times \left(\beta_{i}\times X_{i}-\gamma_{i}\times GB\right),i=1,2,\ldots ,n\text{.} \end{array}$$

A different procedure is used to create the fourth seed to execute the mutation phase in the position updates of the eligible seeds in the search space. The seed's location is updated depending on specific random changes in the selected decision factors. The fourth seed can represent as follows:

(8) $$\begin{array}{c} {Seed}_{i}^{4}=X_{i}\left(x_{i}^{k}=x_{i}^{k}+R\right),k=\left[1,2,\ldots ,d\right]\text{.} \end{array}$$

where *k* is a uniformly distributed random number in the range [0,1], and *R* is a random integer between 1 and *d*. Four possible formulations for *α*_*i*_, which regulate the mobility limits of the seeds, are offered to control and adapt the exploration and exploitation pace of the CGO algorithm:

(9) $$\begin{array}{c} \alpha_{i}=\left\{\begin{array}{c} \text{Rand},\\ 2\times \text{Rand},\\ \left(\delta \times \text{Rand}\right)+1\text{,}\\ \left(\varepsilon \times \text{Rand}\right)+\left(\varepsilon \right)\text{.} \end{array}\right. \end{array}$$

**Figure 1:**
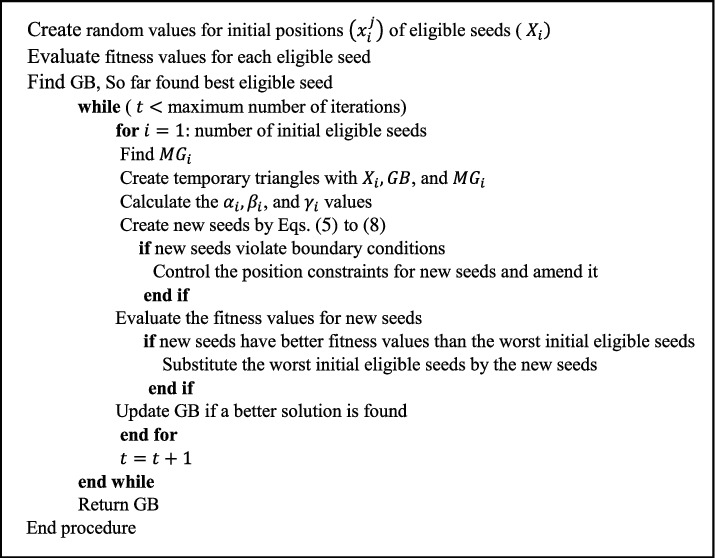
The pseudocode of the CGO algorithm [47].

Rand is a uniformly distributed random number between 0 and 1, while *δ* and *ε* are random integers between 0 and 1. The CGO algorithm's step-by-step approach is listed below, and the algorithm's pseudocode is shown in Figure 1.

### 3.2. SNS

The SNS method was proposed by Bayzidi et al. [48]. This algorithm is based on social networks, which are online platforms that allow people to connect electronically with one another. Users may learn about their favorite people's beliefs and opinions by following them on social media. Consequently, connecting with other network users may impact their opinions. Users are always looking for strategies to increase their network popularity, ensuring that connecting with and influencing other users goes smoothly. Almost every metaheuristic algorithm employs a collection of procedures to generate new solutions. The SNS algorithm generates a novel solution by employing one of four moods that resemble real-world social behavior. The followings are the explanations and mathematical models for these operators (moods) [50].

#### 3.2.1. Mood 1: Imitation

Users may follow each other on social media, and when one publishes a new post, their followers will be notified about the shared subject. Networks have become vital instruments for sharing information and ideas due to this property (propagation of views). Users of social media networks can keep track of their loved ones and celebrities. They will be aware of other people's responses if they follow the news. They will attempt to start a debate about the new event by imitating the viewpoint of another person if the new occurrence involves complex thoughts. This sentiment may be mathematically stated as follows [50]:

(10) $$\begin{array}{c} \begin{array}{c} X_{i\text{new}}=X_{j}+\text{rand}\left(-1,1\right)\times R\text{,}\\ r=X_{j}-X_{i}\text{,}\\ R=rand\left(0,1\right)\times r\text{.} \end{array} \end{array}$$

#### 3.2.2. Mood 2: Conversation

Users of social networks may electronically connect and discuss various topics. Individuals learn from one another and expand their understanding of events during the Talk, which takes the shape of private conversation. Users in conversation have a new perspective on events by hearing other people's perspectives. Lastly, because of the variations in ideas, they may construct a different picture of the problem, according to the following equation [50]:

(11) $$\begin{array}{c} \begin{array}{c} X_{i\text{new}}=X_{k}+R\text{,}\\ D=sign\left(f_{i}-f_{j}\right)\times \left(X_{j}-X_{i}\right)\text{,}\\ R=rand\left(0,1\right)\times D\text{.} \end{array} \end{array}$$

#### 3.2.3. Mood 3: Disputation

Users discuss and defend their opinions on current events to others when in a disputation mood. This work is done on social media sites, such as in the comments and groups. Users may see various points of view from others in the comment area, which may be impacted by the stated reasons. Furthermore, users may form friendly relationships with one another, so they form a virtual group to share their viewpoints on a specific topic. A random group of persons is identified as a commentator or a member of a group to model this attitude, and the new affected perspective in the argument is as follows (12) [50]:

(12) $$\begin{array}{c} X_{inew\;}=X_{i}+rand\left(0,1\right)\times \left(M-AF\times X_{i}\right)\text{,}\\ M=\frac{\overset{N_{r}}{\underset{t}{\sum }}\;X_{t}}{N_{r}}\\ AF=1+round\left(rand\right)\text{.} \end{array}$$

#### 3.2.4. Mood 4: Innovation

Users' ideas and experiences are sometimes reflected in what they post. To put it another way, when a person considers a situation, he or she may be able to see it in a new light and get a better understanding of the problem's nature or gain a whole new perspective on it. A topic might have a variety of characteristics, each of which impacts the problem's comprehension. Consequently, modifying one of their ideas will affect the whole notion of the issue, resulting in a unique viewpoint. This notion is used to generate a new viewpoint via the innovation mood:

(13) $$\begin{array}{c} x_{inew}^{d}=t\times x_{j}^{d}+\left(1-t\right)\times n_{new}^{d},\\ n_{new}^{d}=lb_{d}+{\text{rand}}_{1}\times \left(ub_{d}-lb_{d}\right),\\ t={\text{rand}}_{2}, \end{array}$$

where *D* is the total number of variables in the problem, and ub_d_ and lb_d_ are the lowest and highest values for the *d*^th^ variable, whereas rand_1_ and rand_2_ are two random integers between 0 and 1. *d* is the *d*^th^ variable selected between 1 and *D*. The term *n*_new_^*d*^ refers to a new viewpoint on the problem's *d*^th^ dimension. *x*_*j*_^*d*^ is an existing notion about the *d*^th^ variable provided by another user (*j*^th^ user who chose randomly and *i* ≠ *j*), which the *i*^th^ user desires to change due to a new thought *n*_new_^*d*^. Finally, as *x*_inew_^*d*^, a new viewpoint of the *dt* *h* dimension will be formed. The dimension *x*(*x*_*i*new_^*d*^) will be formed as an interpolation dimension (*x*_*i*new_^*d*^) is a dialogue between the new notion (*n*_new_^*d*^) and the current (*x*_*j*_^*d*^). Changing one dimension, *x*_*i*new_^*d*^ creates a broad shift in the underlying notion and may be considered a new communication point of view. The following model may be used to represent this process:

(14) $$\begin{array}{c} X_{inew}=\left[x_{1},x_{2},x_{3},\ldots x_{i\text{ new}}^{d}\ldots x_{D}\right]\text{.} \end{array}$$

**Figure 2:**
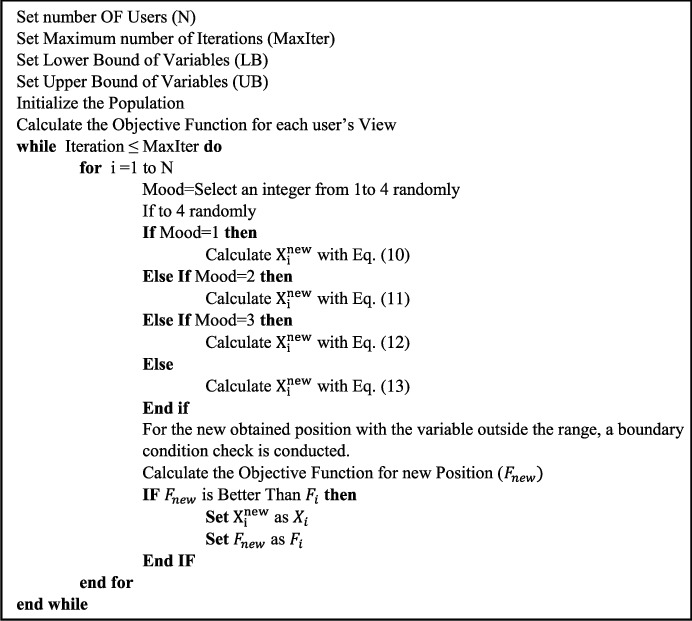
The pseudocode of the SNS algorithm.

The SNS algorithm's step-by-step method is presented in Figure 2, along with the algorithm's pseudocode.

## 4. Numerical Examples

The CGO and SNS are used in this part to solve discontinuous and continuous optimization benchmark problems using two well-known truss designs. Four well-known planar truss structures were tested to ensure that the findings were accurate and that the suggested algorithms were successful. To do the structural analysis, get the members' forces and node displacements, and optimize by using the CGO and SNS techniques, a program was built in the MATLAB software [51] programming environment. All runs are performed on a 64 bit computer with an Intel i7 (2.6 GHz) processor and 12 GB of RAM. The population sizes of CGO and SNS were assumed to be 150 for the truss examples.

### 4.1. 6-Node Planar Truss (10 Members)

**Figure 3:**
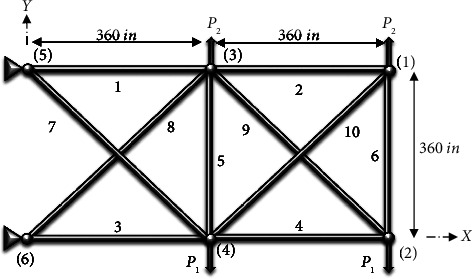
Six-node planar truss.

In this study, the best configuration of a 10-bar planar truss is investigated, as shown in Figure 3.  Table 1 contains the information that may be obtained on the design variables (nd = 10). The number of degrees of freedom (DOF) in this case is eight. This truss is classified into three sizes: loaded, discontinuous, and continuous in the following manner,

#### 4.1.1. Case 1

Loads of *p*_1_ and *p*_2_ are 100 (kips) and zero (kips), respectively, and the sections used to create this truss are as follows:

(15) $$\begin{array}{c} \begin{array}{c} X=\left\{\begin{array}{c} 4.8,4.59,4.49,4.47,4.22,4.18,3.88,3.87,3.84,3.63,3.55,3.38,3.13,3.09,2.93,2.88,2.63,2.62,2.38,2.13,1.99\text{,}\\ 1.8,1.62,33.5,30,26.5,22.9,22,19.9,18.8,16.9,16,15.5,14.2,13.9,13.5,11.5,7.97,7.22,5.74,5.12,4.97 \end{array}\right\}\left({in}^{2}\right) \end{array}\text{.} \end{array}$$

**Figure 4:**
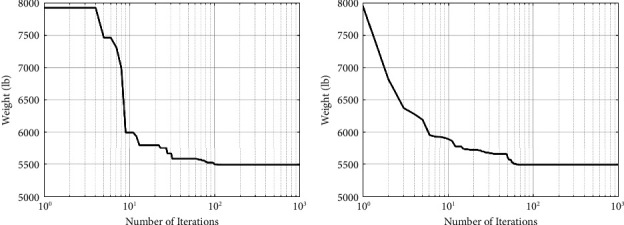
Weight loss process for 10-bar truss—case 1 using (a) SNS and (b) CGO.

**Figure 5:**
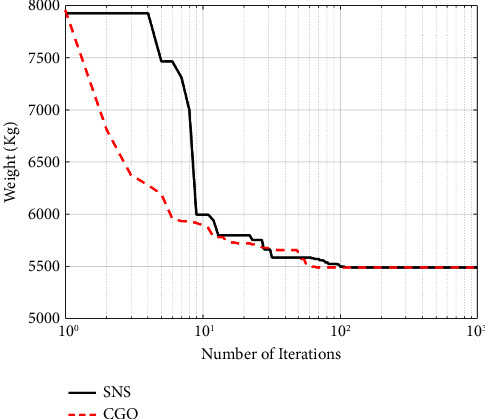
Comparison of 10-bar truss convergence rates—case 1.

The best designs presented so far are listed in Table 2, along with the study's results. As shown in Table 2, the SNS method yields significantly less weight than the CGO, and other algorithms are utilized by other researchers. Figures 4 and 5 depict the path of convergence of the CGO and the SNS algorithms for the six-node planar truss—case1. During the algorithm's execution, these diagrams show the structural weight loss process using the number of function evaluation (NFE).

In the discontinuous size situation, the outputs of the SNS algorithm are more cost-effective than the CGO algorithm, as shown in Figure 5. In addition, the statistical results of the SNS algorithm regarding average and standard deviation are significantly better than the CGO for the discontinuous size case. However, regarding analysis time (CPU) and convergence rate, the CGO is better than the SNS algorithm for the discontinuous size case.

#### 4.1.2. Case 2

Loads of *p*_1_ and *p*_2_ are 100 (“kips”) and zero (“kips”), respectively, and the structure's members are chosen from the series' (*X*) interval as follows:

(16) $$\begin{array}{c} 0.1\leq X\leq 35\left({in}^{2}\right)\text{.} \end{array}$$

**Figure 6:**
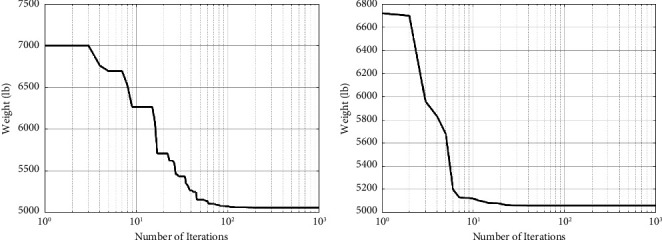
Weight loss process for 10-bar truss—case 2 using (a) SNS and (b) CGO.

**Figure 7:**
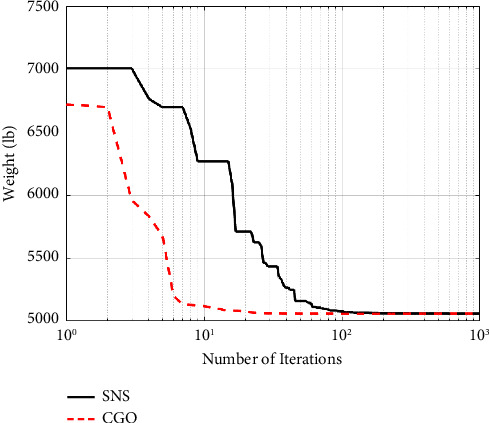
Comparison of 10-bar truss convergence rates—case 2.

The study's conclusions are shown along with a summary of the best designs that have been made so far in Table 3. The quantity of weight acquired from the CGO method is significantly smaller than that obtained from the SNS algorithm and other algorithms, as shown in Table 3. Figures 6 and 7 depict the path of convergence of the CGO and the SNS algorithms for the six-node planar truss—case 2. During the execution of the algorithm, these diagrams show the structural weight loss process using NFE.

The outputs of the CGO algorithm are more cost-effective than the SNS method in the continuous-size situation, as shown in Figure 7. Furthermore, for the continuous-size situation, the statistical results of the CGO algorithm were superior to the SNS method regarding average and standard deviation, as well as analysis time (CPU) and convergence rate.

#### 4.1.3. Case 3

Loads of *p*_1_ and *p*_2_ are 100 (“kips”) and 50 (“kips”), respectively, and the structure's members are chosen from the series' (*X*) interval as follows:

(17) $$\begin{array}{c} 0.1\leq X\leq 35\left({in}^{2}\right)\text{.} \end{array}$$

**Figure 8:**
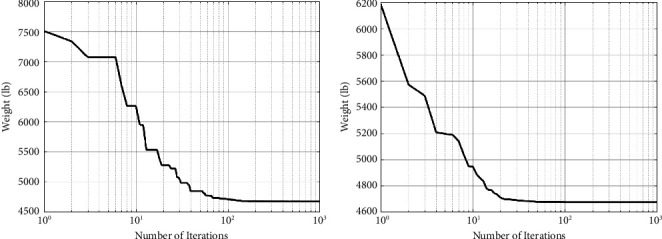
Weight loss process for 10-bar truss—case 3 with (a) SNS and (b) CGO.

**Figure 9:**
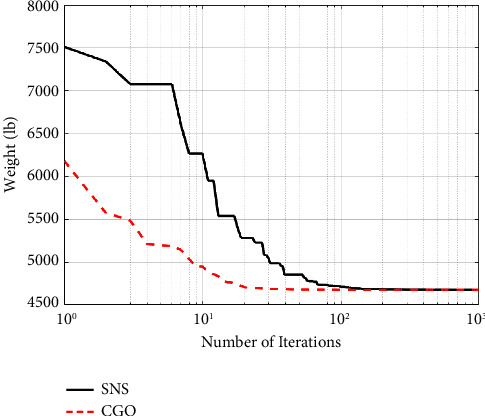
Comparison of 10-bar truss convergence rates—case 3.

Table 4 summarizes the finest ideas provided so far and the study findings. The CGO technique yields considerably less weight than the SNS algorithm and other algorithms, as seen in Table 4. Figures 8 and 9 depict the convergence paths of the CGO and SNS algorithms for the six-node planar truss—case 3. The structural weight reduction process in this form is tracked using the number of function evaluations, which demonstrates that the SNS and CGO are superior to other approaches in this regard.

The outputs of the CGO algorithm are more cost-effective than the SNS method in the continuous-size situation, as shown in Figure 9. Furthermore, for the continuous-size situation, the statistical results of the CGO algorithm were superior to the SNS method regarding average and standard deviation, analysis time (CPU), and convergence rate.

### 4.2. 8-Node Planar Truss (15 Members)

**Figure 10:**
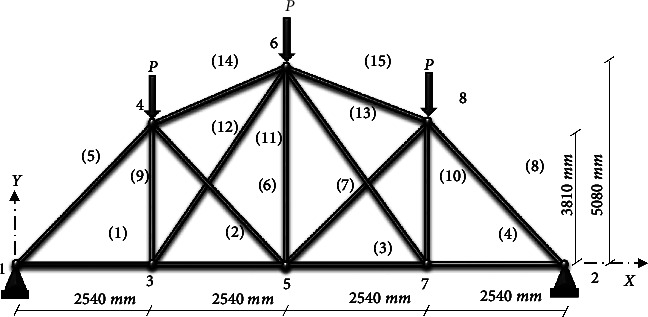
Eight-node planar truss.

The second example of size optimization is a truss system with 15-bar, which is shown in Figure 10. The design factors (nd = 15) are listed in Table 5. The following structural members are selected from the collection of section lists (*X*) for the design of the eight-node planar truss as follows:

(18) $$\begin{array}{c} Χ=\left\{113.2,143.2,145.9,174.9,185.9,235.9,265.9,297.1,308.6,334.3,338.2,497.8,507.6,736.7,791.2,1063.7\right\}\left({mm}^{2}\right) \end{array}$$

**Figure 11:**
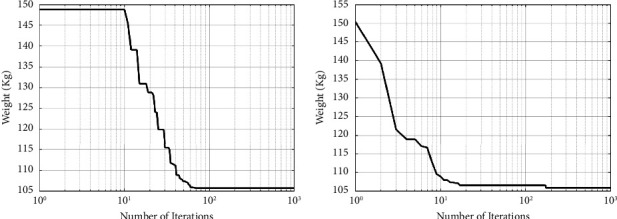
Weight loss process for 15-bar truss using (a) SNS and (b) CGO.

A maximum number of 1000 iterations were set for comparison with other algorithms in the same circumstance.  Table 6 compares the optimum design of CGO and SNS to PSO [34], PSOPC [34], HPSO [34], WCA [39], and MBA [33].  Table 6 shows that SNS achieved an optimum design value comparable to or better than other methods, whereas, in terms of the number of analyses, SNS and CGO are better than other methods.  Figure 11 also shows the convergence route of the CGO and the SNS algorithms for an eight-node planar truss.

**Figure 12:**
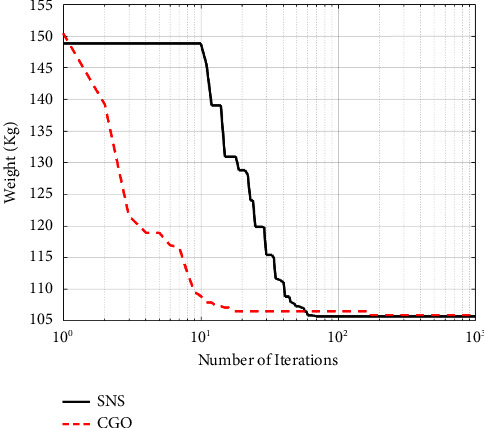
Comparison of convergence curves of SNS and CGO algorithms for the 15-bar problem.

As shown in Figure 12, in the discontinuous-size situation, the outputs of the SNS algorithm are more cost-effective than the CGO method. The statistical results of the SNS algorithm regarding average and standard deviation are significantly better than the CGO for the discontinuous size case. However, the CGO outperforms the SNS algorithm regarding analysis time (CPU) and convergence rate.

### 4.3. 9-Node Planar Truss (17 Members)

**Figure 13:**
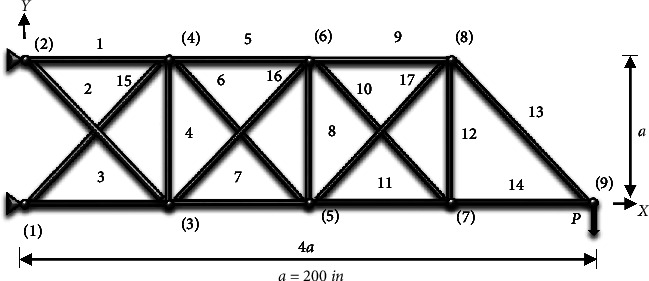
Nine-node planar truss.

The optimal design of a 17-bar planar truss is explored, as illustrated in Figure 13. The information about the design variables (nd = 17) is presented in Table 7.

The cross-sectional areas of components are regarded as 17 sizing design factors, with the following minimum permissible values:

(19) $$\begin{array}{c} 0.1\leq X\left({in}^{2}\right)\text{.} \end{array}$$

**Figure 14:**
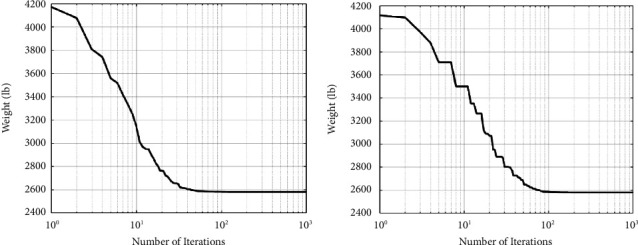
Weight loss process for 17-bar truss using (a) SNS and (b) CGO.

**Figure 15:**
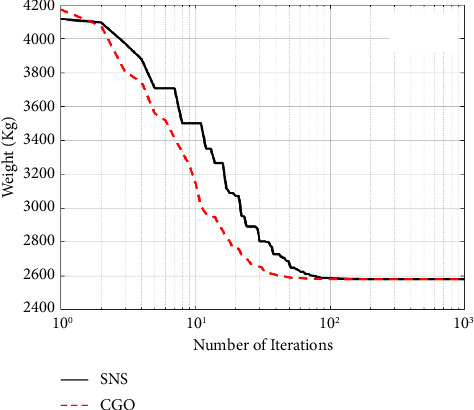
Comparison of convergence curves of SNS and CGO algorithms for the 17-bar problem.

Table 8 contains an overview of the finest designs that have been provided so far, as well as the findings of this study. The amount of weight acquired from the CGO method is significantly smaller than the amount obtained from the SNS algorithm and other algorithms, as shown in Table 8. Figures 14 and 15 demonstrate the convergence paths of the CGO and SNS algorithms for a nine-node planar truss. The NFE is used to monitor the structural weight reduction process in this form, which shows that the SNS and CGO in this term are better than other methods.

As shown in Figure 15, the outputs of the CGO method are more cost-effective than the SNS algorithm in the continuous-size situation. Also, the CGO algorithm got better statistical results for the continuous-size situation in terms of average, standard deviation, analysis time (CPU), worst weight, and convergence rate than the SNS technique.

### 4.4. 20-Node Planar Truss (45 Members)

**Figure 16:**
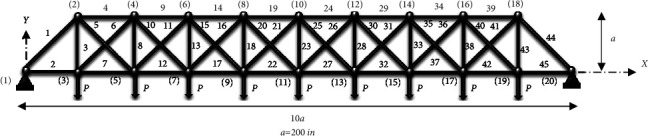
Twenty-node planar truss.

The optimal design of a 45-bar planar truss, as illustrated in Figure 16, is evaluated in the last case. The design factors (nd = 45) are listed in Table 9.

For design reasons, the structure's components are divided into 23 groups according to Table 10, taking into account the symmetry of the structure. There is a lower limit for each size variable:

(20) $$\begin{array}{c} 0.1\leq X\left({in}^{2}\right)\text{.} \end{array}$$

**Figure 17:**
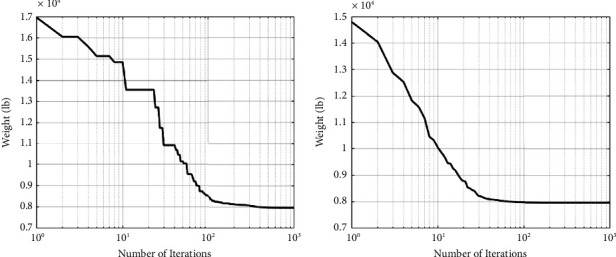
Weight loss process for the 45-bar truss using (a) SNS and (b) CGO.

**Figure 18:**
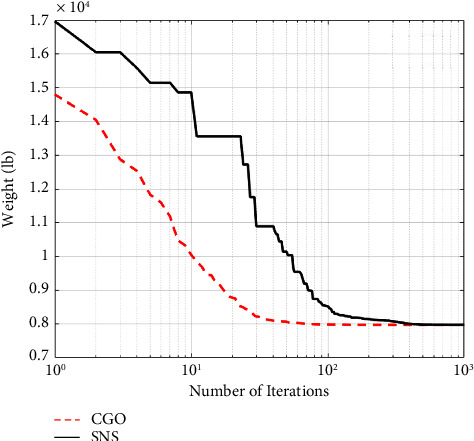
Comparison of convergence curves of SNS and CGO algorithms for the 45-bar problem.

Table 10 contains a summary of the finest ideas provided so far, as well as the findings of this study. As shown in Table 10, the amount of weight gained by the CGO method is much less than that obtained by the SNS algorithm and other methods.  Figure 17 and Figure 18 demonstrate the route of convergence of the CGO method and the SNS algorithm for the twenty-node planar truss. The NFE is used to examine the structural weight reduction process in this form, which shows that the SNS and CGO in this term are better than other methods.

As shown in Figure 18, the outputs of the CGO algorithm are more cost-effective than the SNS method in the continuous-size situation. Additionally, for continuous-size cases, the CGO algorithm's statistical findings outperform the SNS approach regarding average and standard deviation, analysis time (CPU), and convergence rate.

### 4.5. Summary Results

To present a better comparison of the CGO and SNS algorithms, Table 11 shows the standard deviation, worst, best, mean, and CPU time of the outcomes. As previously stated, both algorithms are run 1500 times using a seed number and a population size of 150.

## 5. Conclusion

The chaos game optimization approach and the social network search algorithm were used to analyze the continuous and discontinuous size optimization of planar trusses in this research. The findings of a computer analysis of four distinct kinds of planar trusses (6-node, 8-node, 9-node, and 20-node) that were evaluated under various degrees of discontinuous and continuous size and loading are presented in this study.

1. The solved cases demonstrate that the chaos game optimization algorithm and the social network search algorithm can solve the continuous and discontinuous size optimization problems and that these algorithms can find the best structural configuration faster than other methods. Other advantages of using the chaos game optimization algorithm with the social network search technique to solve structural optimization problems include a faster convergence rate, a better solution, and low computational effort.
2. In discontinuous sizes, the designs generated by the social network search algorithm are much more cost-effective than other designs, whereas, in continuous-size instances, the designs generated by the chaos game optimization technique are far more cost-effective.
3. According to a study of convergence rate diagrams in terms of CPU time and function evaluations, the chaos game optimization technique approaches the optimal solution faster than the social network search method.

## Figures and Tables

**Table 1:** Design data for the 10-bar planar truss.

Material properties		

Elastic modulus (*E*) = 10000ksi density (*ρ*) = 0.1lb/in^3^		

Constraint data		

Displacement constraints:		
In the direction of *X*-axis and *Y*-axis |Δ_*j*_| ⩽ 2 in (50.8 mm)*j*=1,2,3,4 Δ_*a*_=±2 in (50.8 mm)		
Stress constraints:		
|*σ*_*i*_| ⩽ 25ksi(172.25Mpa)*i*=1,2,…, 10 *σ*_*a*_=±25ksi(172.25Mpa)		
Loading data		

Case 1	Nodal number	P_y_(kips)
	2	−100(−445.374kN)
	4	−100(−445.374kN)

Case 2	Nodal number	P_y_(kips)
	2	−100(−445.374kN)
	4	−100(−445.374kN)

Case 3	Nodal number	P_y_(kips)
	1, 3	150(445.374kN)
	2, 4	−50(−445.374kN)

**Table 2:** Comparison of optimal results obtained for the six-node planar truss—case 1.

Design variables (in^2^)	Sadollah et al. [33]	Li et al. [34]			Ho-Huu et al. [36]		Rajeev et al. [17]	Present study	
	MBA	PSO	PSOPC	HPSO	aeDE	DE	GA	CGO	SNS
*X*1	30	30	30	30	33.5	33.5	33.5	33.5	33.5
*X*2	1.62	1.62	1.8	1.62	1.62	1.62	1.62	1.62	1.62
*X*3	22.9	30	26.5	22.9	22.9	22.9	22	22.9	22.9
*X*4	14.2	13.5	15.5	13.5	14.2	14.2	15.5	15.5	14.2
*X*5	1.62	1.62	1.62	1.62	1.62	1.62	1.62	1.62	1.62
*X*6	1.62	1.8	1.62	1.62	1.62	1.62	1.62	1.62	1.62
*X*7	7.97	11.5	11.5	7.97	7.97	7.97	14.2	7.97	7.97
*X*8	22.9	18.8	18.8	26.5	22.9	22.9	19.9	22	22.9
*X*9	22.9	22	22	22	22	22	19.9	22	22
*X*10	1.62	1.8	3.09	1.8	1.62	1.62	2.62	1.62	1.62

Best weight (lb)	5507.75	5581.76	5593.44	5531.98	5490.74	5490.74	5613.84	5491.72	5490.74
Average weight (lb)	5527.296	N/A	N/A	N/A	5502.623	5501.547	N/A	5521.81	5495.36
Worst weight (lb)	5536.965	N/A	N/A	N/A	5549.204	5546.685	N/A	5541.94	5536.97
Std dev (lb)	11.38	N/A	N/A	N/A	20.780	19.521	N/A	26.01	12.99
Number of analysis	3600	N/A	N/A	N/A	2380	6440	N/A	1500	1500
CPU time (min)	N/A	N/A	N/A	N/A	N/A	N/A	N/A	0.58	1.37

**Table 3:** Comparison of optimal results obtained for the six-node planar truss—case 2.

Design variables (in^2^)	Farshi and Alinia-Ziazi [37]	Li et al. [35]	Hadidi et al. [38]	Eskandar et al. [39]	Sangtarash et al. [40]	Kooshkbaghi and Kaveh [41]	Kaveh and Malakoutirad [42]	Present study	
	CaF	HPSO	ABC	WCA	HPBA	ACCS	HGAPSO	CGO	SNS
*X*1	30.52	30.7	34.31	29.5	30.36	30.64	30.63	30.56	30.52
*X*2	0.1	0.1	0.1	0.1	0.1	0.1	0.1	0.1	0.10
*X*3	23.20	23.17	20.67	24	23.80	23.1	23.06	23.14	23.13
*X*4	15.22	15.18	14.51	15	14.80	15.06	15.01	15.21	15.19
*X*5	0.1	0.1	0.1	0.1	0.1	0.1	0.1	0.1	0.10
*X*6	0.55	0.55	0.66	0.5	0.56	0.57	0.59	0.55	0.55
*X*7	7.47	7.46	7.87	7.5	7.42	7.48	7.49	7.47	7.47
*X*8	21.03	20.98	20.35	21	21.12	21.09	21.1	21.06	21.09
*X*9	21.53	21.51	22.02	22	21.47	21.53	21.56	21.52	21.53
*X*10	0.1	0.1	0.1	0.1	0.1	0.1	0.1	0.1	0.10

Best weight (lb)	5061.4	5060.92	5095.33	5067.33	5062.01	5061.03	5061.4	5060.85	5060.89
Average weight (lb)	N/A	N/A	5113.92	N/A	5062.19	5061.07	N/A	5060.87	5060.96
Worst weight (lb)	N/A	N/A	5187.19	N/A	N/A	N/A	N/A	5060.97	5061.15
Std dev (lb)	N/A	N/A	24.793	N/A	0.26	0.09	N/A	0.03	0.06
Number of analysis	N/A	125000	N/A	N/A	8000	12000	N/A	1500	1500
CPU time (min)	N/A	N/A	N/A	N/A	N/A	N/A	N/A	0.76	1.27

**Table 4:** Comparison of optimal results obtained for the 6-node planar truss—case 3.

Design variables (in^2^)	Khan et al. [43]	Li et al. [35]	Venkayya [52]	Hadidi et al. [38]		Sangtarash et al. [40]	Present study	
	GA	HPSO	SEC	ABC	MABC	HPBA	CGO	SNS
*X*1	24.72	23.35	25.19	24.81	23.64	24.00	23.53	23.54
*X*2	0.1	0.1	0.363	0.1	0.1	0.1	0.1	0.1
*X*3	26.54	25.5	25.42	26.05	26.32	25.13	25.28	25.22
*X*4	13.22	14.25	14.33	14.88	14.41	14.33	14.37	14.35
*X*5	0.11	0.1	0.417	0.1	0.1	0.1	0.1	0.1
*X*6	4.84	1.97	3.144	2.01	1.97	1.974	1.97	1.97
*X*7	12.66	12.36	12.08	12.45	12.38	12.52	12.39	12.41
*X*8	13.78	12.89	14.61	12.68	12.77	12.98	12.83	12.85
*X*9	18.44	20.36	20.26	18.87	20.27	19.88	20.33	20.34
*X*10	0.1	0.1	0.513	0.1	0.1	0.1	0.1	0.1

Best weight (lb)	4792.52	4677.29	4895.60	4691.07	4677.06	4678.11	4676.92	4676.97
Average weight (lb)	N/A	N/A	N/A	4708.57	4677.74	4678.48	4676.93	4677.18
Worst weight (lb)	N/A	N/A	N/A	4753.2	4679.52	N/A	4676.96	4677.63
Std dev (lb)	N/A	N/A	N/A	16.738	0.725	0.301	0.01	0.16
Number of analysis	N/A	125000	N/A	N/A	N/A	8000	1500	1500
CPU time (min)	N/A	N/A	N/A	N/A	N/A	N/A	0.98	1.37

**Table 5:** Design data for the 15-bar planar truss problem.

Material properties	

Elastic modulus (*E*) = 200 *GPa* density(*ρ*) = 7800*kg*/*m*^3^	

Constraint data	

Displacement constraints:	
In the direction of *X*-axis and *Y*-axis |Δ_*j*_| ⩽ 10 *mmj*=3,4,5,6,7,8Δ_*a*_=10 *mm*	
Stress constraints:	
|*σ*_*i*_| ⩽ 120*Mpa* *i*=1,2,…, 15*σ*_*a*_=±120*Mpa*	

Loading data	

Nodal number	*P*_*y*_(kN)

4	−35
6	−35
8	−35

**Table 6:** Comparison of optimal results obtained for the 8-node planar truss problem.

Design variables (in^2^)	Yang et al. [44]	Li et al. [34]			Eskandar et al. [39]	Sadollah et al. [33]	This study	
	CPSO3	PSO	PSOPC	HPSO	WCA	MBA	CGO	SNS
*X*1	113.2	185.9	113.2	113.2	113.2	113.2	113.2	113.2
*X*2	113.2	113.2	113.2	113.2	113.2	113.2	113.2	113.2
*X*3	113.2	143.2	113.2	113.2	113.2	113.2	113.2	113.2
*X*4	113.2	113.2	113.2	113.2	113.2	113.2	113.2	113.2
*X*5	736.7	736.7	736.7	736.7	736.7	736.7	736.7	736.7
*X*6	113.2	143.2	113.2	113.2	113.2	113.2	113.2	113.2
*X*7	113.2	113.2	113.2	113.2	113.2	113.2	113.2	113.2
*X*8	736.7	736.7	736.7	736.7	736.7	736.7	736.7	736.7
*X*9	113.2	113.2	113.2	113.2	113.2	113.2	113.2	113.2
*X*10	113.2	113.2	113.2	113.2	113.2	113.2	113.2	113.2
*X*11	113.2	113.2	113.2	113.2	113.2	113.2	113.2	113.2
*X*12	113.2	113.2	113.2	113.2	113.2	113.2	113.2	113.2
*X*13	113.2	113.2	113.2	113.2	113.2	113.2	113.2	113.2
*X*14	334.3	334.3	334.3	334.3	334.3	334.3	338.2	334.3
*X*15	334.3	334.3	334.3	334.3	334.3	334.3	338.2	334.3

Best weight (kg)	105.735	108.84	108.96	105.735	105.735	105.735	105.735	105.735
Average weight (kg)	N/A	N/A	N/A	N/A	N/A	N/A	106.2	105.735
Std Dev (kg)	N/A	N/A	N/A	N/A	N/A	N/A	0.36	0
Worst weight (kg)	N/A	N/A	N/A	N/A	N/A	N/A	106.42	105.735
Number of analysis	12,500	N/A	N/A	N/A	1700	N/A	1500	1500
CPU time (min)	N/A	N/A	N/A	N/A	N/A	N/A	0.95	1.52

**Table 7:** Design data for the 17-bar planar truss problem.

Material properties	

Elastic modulus (E) = 30000*ksi* density (*ρ*) = 0.268lb/*in*^3^	

Constraint data	

Displacement constraints:	
In the direction of *X*-axis and *Y*-axis |Δ_*j*_| ⩽ 2 in (50.8 *mm*) *j*=3,4,5,6,7,8,9Δ_*a*_=±2 in (50.8 *mm*)	
Stress constraints:	
|*σ*_*i*_| ⩽ 50 *ksi*(344.5*Mpa*) *i*=1,2,…, 17*σ*_*a*_=±50*ksi*(344.5*Mpa*)	

Loading data	

Nodal number	*P*_*y*_(kips)

9	−100(−445.374kN)

**Table 8:** Comparison of optimal results obtained for the 9-node planar truss problem.

Design variables (in^2^)	Sangtarash et al. [40]		Kazemzadeh Azad and Hasancebi [45]	Kaveh and Ghazaan [46]	Sangtarash et al. [40]	This study	
	BB-BC	HPBA	ESASS	ECBO	APO	CGO	SNS
*X*1	14.4156	15.8	15.9324	15.9158	16	15.7022	15.9328
*X*2	0.515	0.11	0.1	0.1001	0.1	0.1	0.11
*X*3	13.1706	12.12	12.0193	12.0762	12.28	11.8988	12.0538
*X*4	0.1034	0.1	0.1	0.1	0.1	0.1	0.1
*X*5	8.8999	8.05	8.1001	8.0527	7.91	7.9534	8.0605
*X*6	5.1549	5.6	5.53	5.5611	5.52	5.4810	5.5785
*X*7	11.4214	11.97	11.9209	11.9470	12.78	11.7617	11.9402
*X*8	0.1101	0.1	0.1	0.1	0.1	0.1	0.1
*X*9	7.9223	7.88	8.0128	7.9425	7.45	7.8306	7.9048
*X*10	0.1782	0.1	0.1	0.1	0.1	0.1	0.1
*X*11	4.4553	4.07	4.0715	4.0589	3.96	3.9984	4.0668
*X*12	0.1389	0.1	0.1	0.1	0.1	0.1000	0.1004
*X*13	5.8455	5.66	5.6726	5.6644	5.82	5.5762	5.6515
*X*14	4.1933	4.05	4.0154	4.0057	3.61	3.9430	3.9883
*X*15	5.1536	5.52	5.5286	5.5565	5.63	5.4775	5.5663
*X*16	0.4065	0.1	0.1	0.1	0.1	0.1	0.1
*X*17	5.4519	5.61	5.5739	5.5740	5.59	5.4979	5.5898

Best weight (lb)	2598.4	2582.00	2581.93	2581.89	2588.98	2581.37	2581.92
Average weight (lb)	2599.03	2582.74	N/A	2597.11	2589.41	2581.87	2581.98
Std dev (lb)	0.542	0.214	N/A	22.41	0.391	0.07	0.18
Worst weight (lb)	N/A	N/A	N/A	N/A	N/A	2581.89	2582.13
Number of analysis	13000	10500	5941	16000	11000	1500	1500
CPU time (min)	N/A	N/A	N/A	N/A	N/A	2.54	3.23

**Table 9:** Design data for the 45-bar planar truss problem.

Material properties	

Elastic modulus (E) = 30000ksi density (*ρ*) = 0.283*lb*/in^3^	

Constraint data	

Displacement constraints:	
In the direction of *X*-axis and *Y*-axis |Δ_*j*_| ⩽ 2 in (50.8 mm) *j*=2 , 3,…, 19Δ_a_=±2 in (50.8 mm)	

Stress constraints:	
|*σ*_*i*_| ⩽ 30 ksi(206.7Mpa) *i*=1,2,…, 45*σ*_a_=±30ksi(206.7Mpa)	

Loading data	
Nodal number	*P*_*y*_(kips)
2,3,…, 19	−100

**Table 10:** Comparison of optimal results obtained for the 20-node planar truss problem.

Design variables (in^2^)	Members	Kazemzadeh Azad and Hasancebi [45]	Hadidi et al. [38]		This study	
		ESASS	ABC	MABC	CGO	SNS
*X*1	1, 44	4.6052	4.5996	4.6052	4.5703	4.5765
*X*2	2, 45	3.7083	3.7966	3.7083	3.7352	3.7455
*X*3	3, 43	3.1919	3.0497	3.1919	3.1634	2.9437
*X*4	4, 39	3.2756	3.2841	3.2756	3.3025	3.5052
*X*5	5, 41	0.1	0.1069	0.1	0.1	0.4
*X*6	6, 40	3.9896	3.9279	3.9896	3.9302	3.6704
*X*7	7, 42	0.8916	0.9649	0.8916	0.9315	1.1441
*X*8	8, 38	1.217	1.2133	1.217	1.2061	1.0314
*X*9	9, 34	7.7323	7.6553	7.7323	7.6830	7.6490
*X*10	10, 36	2.2227	2.1993	2.2227	2.1819	2.1760
*X*11	11, 35	1.1803	1.1929	1.1803	1.2128	1.2214
*X*12	12, 37	0.1	0.1001	0.1	0.1	0.1
*X*13	13, 33	0.1	0.1008	0.1	0.1	0.1
*X*14	14, 29	9.3901	9.536	9.3901	9.4270	9.4293
*X*15	15, 31	1.2149	1.2173	1.2149	1.2164	1.2267
*X*16	16, 30	1.3322	1.419	1.3322	1.3612	1.3667
*X*17	17, 32	2.6056	2.5513	2.6056	2.5865	2.5936
*X*18	18, 28	0.1	0.1	0.1	0.1	0.1
*X*19	19, 24	11.6266	11.5439	11.6266	11.6804	11.6775
*X*20	20, 26	1.2406	1.2807	1.2406	1.2726	1.2733
*X*21	21, 25	0.1	0.101	0.1	0.1	0.1
*X*22	22, 27	3.7923	3.7598	3.7923	3.7639	3.7523
*X*23	23	0.1	0.1017	0.1	0.1	0.1

Best weight (lb)		7967.98	7968.95	7967.98	7966.96	7968.78
Average weight (lb)		N/A	8472.46	8030.78	7968.09	7969.43
Worst weight (lb)		N/A	8690.35	8230.11	7968.69	7970.05
Std Dev (lb)		N/A	101.929	98.507	0.48	0.87
Number of analysis		9349	N/A	N/A	1500	1500
CPU time (min)		N/A	N/A	N/A	5.21	8.02

**Table 11:** Summary results for the CGO and SNS algorithms.

Example		Number of analysis		Best weight (lb)		Worst weight (lb)		Mean weight (lb)		Standard deviation (lb)		CPU time (min)	
		CGO	SNS	CGO	SNS	CGO	SNS	CGO	SNS	CGO	SNS	CGO	SNS
6-node (10 members)	Case 1	1500	1500	5491.72	5490.74	5541.94	5536.97	5521.81	5495.36	26.01	12.99	0.58	1.37
	Case 2	1500	1500	5060.85	5060.89	5060.97	5061.15	5060.87	5060.96	0.03	0.06	0.76	1.27
	Case 3	1500	1500	4676.92	4676.97	4676.96	4677.63	4676.93	4677.18	0.01	0.16	0.98	1.37
8-node (15 members)		1500	1500	105.735	105.735	106.42	105.735	106.2	105.735	0.36	0	0.95	1.52
9-node (17 members)		1500	1500	2581.37	2581.92	2581.89	2582.13	2581.87	2581.98	0.07	0.18	2.54	3.23
20-node (45 members)		1500	1500	7966.96	7968.78	7968.69	7970.05	7968.09	7969.43	0.48	0.87	5.21	8.02

## Data Availability

No data were used to support this study.

## Nomenclature

CGO: Chaos game optimization
SNS: Social network search.
